# Effect of Probiotic Supplementation on Gut Microbiota in Children with Autism: A Pilot Randomised Controlled Trial

**DOI:** 10.3390/nu18132079

**Published:** 2026-06-25

**Authors:** Sachin Agrawal, Shripada Rao, Andrew Whitehouse, Gail A. Alvares, Alpana Kulkarni, Jessica A. Taylor, Patricia L. Conway, Torsten Thomas, Sanjay Patole

**Affiliations:** 1 Neonatal Directorate, King Edward Memorial Hospital for Women, Perth, WA 6008, Australia; sanjay.patole@health.wa.gov.au; 2 School of Medicine, The University of Western Australia, Perth, WA 6009, Australia; shripada.rao@health.wa.gov.au; 3 Perth Children’s Hospital, Perth, WA 6009, Australia; 4 The Kids Research Institute Australia, Nedlands, WA 6009, Australia; andrew.whitehouse@thekids.org.au (A.W.); gail.alvares@thekids.org.au (G.A.A.); 5 UWA Centre for Child Health Research, The University of Western Australia, Perth, WA 6009, Australia; 6 Fremantle Child Development Service, Perth, WA 6163, Australia; alpana.kulkarni@health.wa.gov.au; 7 PC Biome, NTU Innovation Centre, Nanyang Technological University, Singapore 638075, Singapore; 8 Centre for Marine Science and Innovation and School of Biological, Earth and Environmental Sciences, University of New South Wales (UNSW), Sydney, NSW 2052, Australia; pconway@ntu.edu.sg (P.L.C.); t.thomas@unsw.edu.au (T.T.); 9 Singapore Centre for Environmental Life Sciences Engineering, Nanyang Technological University, Singapore 637551, Singapore

**Keywords:** autism spectrum disorder, behavior, microbiota, probiotics

## Abstract

Background: Dysbiosis of the gut microbiota is common in children with autism spectrum disorder (ASD). Probiotics have the potential to improve outcomes in ASD by modulating the gut microbiota–brain axis. Methods: In a pilot randomised trial, children (2 to 5 years) with confirmed ASD (DSM-5 criteria) received either a multi-strain probiotic (450 billion CFU twice daily for one month, followed by once daily for three months) or placebo supplementation. Faecal microbiota profiles were assessed using pre- and post-supplementation samples. The primary outcome involved changes in gut microbiota diversity. Secondary outcomes included faecal short-chain fatty acid levels and behavioural changes. Results: Difficulties in recruitment and loss to follow-up for reasons including COVID-19 resulted in the enrolment of only 23 (probiotic: 9; placebo: 14) instead of the planned 40 children. There was no evidence of changes in the gut microbiota in probiotic-supplemented children. The common phyla were Bacillota_A (~50%), Bacteroidota (~18%) and Actinobacteriota (~10%). Alpha- and Beta-diversity showed no significant difference between pre- vs. post-supplementation samples. Bifodobacteriaceae increased significantly in the probiotic-supplemented group (*p* = 0.046). Conclusions: The increase in faecal Bifodobacteriaceae supports an evaluation of probiotics in ASD. Addressing the reasons for loss to follow-up is important when designing trials in this field.

## 1. Introduction

The term autism spectrum disorder (ASD) covers a set of heterogeneous neurodevelopmental conditions, characterised by early-onset difficulties in social communication and unusually restricted, repetitive behaviour and interests [1]. The prevalence of ASD has increased considerably over the past three decades, and is currently diagnosed in 1.46% of individuals [2]. The estimated lifelong cost of care for an ASD patient is $2.4 million in the USA and £1.5 million in the UK [3]. A Western Australian study reported the median ASD-related annual cost to the family of an individual with ASD as $34,900, with an extra cost of $1400/year for each additional symptom [4]. The pathogenesis of ASD is complex and poorly understood, involving genetic predisposition and a range of environmental factors, including intestinal dysbiosis, excessive inflammation, altered intestinal permeability and immune imbalance [5]. Limited options are currently available for improving outcomes for children with ASD. Behavioural interventions and drugs improve ASD symptoms only partially, and drugs are often associated with adverse effects [6,7]. Furthermore, a systematic review has found no conclusive evidence supporting the role of complementary and alternative therapies for improving ASD symptoms [8]. Gut symptoms are common in children with ASD. The median (range) prevalence of constipation, diarrhoea, and any or ≥ one symptom in ASD is 22% (4.3–45.5%), 13.0% (2.3–75.6%), and 46.8% (4.2–96.8%), respectively [9]. A systematic review showed that gut dysbiosis is common in children with ASD [10,11,12,13]. Various investigators have reported that disruption of the gut–brain axis [14] and elevated short-chain fatty acids (SCFA), especially propionic acid [15,16], may play an important role in the pathogenesis of ASD. For example, Fiorentino et al. reported increased expression of genes and proteins associated with blood–brain barrier dysfunction, neuro-inflammation, and intestinal permeability in post-mortem brain and duodenal samples from children with versus without ASD or Schizophrenia [17]. Furthermore, the promising results of faecal microbiota transplant support the involvement of gut microbiota in ASD [18]. Emerging evidence suggests that modulating the gut microbiota–brain axis by probiotic supplementation may be a novel strategy for improving developmental outcomes in ASD [19,20]. The beneficial effect of probiotics relates to modulation of the gut microbiota–brain axis through various pathways, including correction of dysbiosis, inhibition of gut colonisation by pathogens, enhancing gut barrier function and enteric nervous system maturation, and exerting an anti-inflammatory effect [21,22,23,24,25,26,27].

A recent updated systematic review and expert recommendations emphasise the need for rigorous randomised controlled trials (RCTs) of probiotic supplementation in children with ASD [28,29,30]. Considering the encouraging evidence in totality, we aimed to evaluate the role of probiotics in children with ASD in a well-designed RCT.

## 2. Aim and Hypothesis

We aimed to evaluate the effect of probiotic supplementation on gut microbiota in a community-based sample of children with autism and assess the feasibility of conducting an adequately powered RCT focussed on clinically important outcomes (e.g., behavioural change) in this population.

**Hypothesis** **1.**  *Children with autism receiving probiotic supplementation will have significant compositional changes in the gut microbiota compared with those receiving a placebo.*

## 3. Methods and Participants

Study Design: This was a pilot double-blind RCT comparing probiotic and placebo supplementation for a duration of four months.

Ethics: Institutional ethics committee approval number RGS00000003318, University of Western Australia HREC-2022/ET000565, Trial Registration: ACTRN12621000029897, Date of registration: 14 January 2021.

Randomisation, Allocation Concealment, and Blinding: Randomisation (using computer-generated random numbers) was stratified based on Mullen’s Early Learning Nonverbal Developmental Quotient, categorised as <85 or ≥85 [31]. Allocation concealment was optimised by using serially numbered, sealed, coded, opaque envelopes. The probiotic and placebo sachets were of equal volume and identical in appearance. All investigators, participants, and outcome assessors were blinded to the allocation status during the trial. Randomisation was conducted when a participant met all eligibility criteria and completed baseline assessments. Once consent was obtained, the trial manager (SA) informed the clinical trial pharmacy, which randomly allocated the participants to coded sachets containing either the probiotic or placebo to maintain blinding [32].

## 4. Participants

Inclusion Criteria: (1) Age: 2–5 years; (2) confirmed diagnosis of autism based on DSM-5 criteria [33].

Exclusion Criteria: (1) Major congenital anomalies; (2) epilepsy syndromes, significant sensory impairment (e.g., blindness, deafness), or neonatal hypoxic–ischemic encephlopathy requiring therapeutic cooling; (3) Coeliac disease or inflammatory bowel disease; (4) use of probiotics for ≥4 weeks in the 90 days before enrolment; (5) current or recent (within 4 weeks before enrolment) exposure to antibiotics, chemotherapy or immunosuppressant agents, or laxatives; (6) prosthetic devices including heart valves; (7) confirmed HIV, Hepatitis B, and/or Hepatitis C; (8) known allergy to probiotics; (9) special diets; (10) cows’ milk protein allergy or food allergy.

Recruitment: Participants were recruited from the community through CliniKids (The Kids Research Institute Australia, Perth, Western Australia). Recruitment was conducted by contacting families who expressed interest in research trials via CliniKids, advertising on social media, and engaging with local service providers and community organisations to promote the study. The period of recruitment was between August 2021 and August 2023.

Intervention: The probiotic selected for this study was Vivomixx^®^ Mendes S.A., Lugano, Switzerland. Each sachet contained a total of 450 billion (450 × 10^9^) lyophilised bacterial cells of eight probiotic strains: *Streptococcus thermophilus* DSM 24731, *Bifidobacterium breve* DSM 24732, *Bifidobacterium longum* DSM 24736, *Bifidobacterium infantis* DSM 24737, and *Lactobacillus acidophilus* DSM 24735, *Lactiplantibacillus plantarum* DSM 24730, *Lacticaseibacillus paracasei* DSM 24733, *Lactobacillus delbrueckii* subsp. *bulgaricus* DSM 24734). The water-soluble probiotic powder was administered orally, either dissolved directly in the mouth or mixed in a cold, non-carbonated drink.

Probiotic Protocol: Participants were assigned to treatment group A (probiotics) or group B (placebo). Treatment group A received 450 billion CFU of probiotics twice daily for one month, followed by 450 billion CFU once daily for three months [34]. Treatment group B participants received placebo sachets with an equal volume containing 4.4 g of maltose and silicon dioxide. Parents were provided with instructions on how to administer the supplementation to their child at home. The four-month duration of supplementation was to ensure adequate time for gut colonisation, which requires 2–3 weeks on average [35]. Probiotic and placebo sachets were kept in a secure fridge using an electronic temperature logging system in the hospital pharmacy (records temperature every two minutes and sends an alarm when temperature exceeds the 2–8-degree C range). The temperature log was monitored weekly to ensure maintenance of environmental conditions. At home, parents were instructed to store the sachets at 2–8 degrees C in the kitchen refrigerator. After completing the trial, parents returned any unused sachets.

Stool Sample Collection and Analysis: Changes in faecal microbiota were assessed using two samples collected from each participant (before starting and after completing 4 months of supplementation). Microbial community analysis was performed by extracting DNA from faecal material and creating an 16S rRNA gene amplicon library, from which microbial community structure and composition was compared between groups.

Outcomes: The primary outcome was “differences in gut microbiota”. Differences were assessed by measuring alpha-diversity indices, similarities of community structure and composition, and the relative abundance of specific microbial taxa.

Secondary outcomes included faecal short-chain fatty acids (SCFA: acetate, propionate, and butyrate) measured before and after 4 months of supplementation.

Clinical outcomes were assessed by Repetitive Behaviour Scale—Revised (RBS-R), The Short Sensory Profile (SSP-2), Vineland Adaptive Behavioural Scales—3rd edition (VABS III), Social Responsiveness Scales (SRS-2), and Australian Eating scale (AES), completed pre- and post-supplementation. Dietary changes were recorded.

Questionnaires were completed by parents at visits pre- and post-the 4 month period of supplementation and returned to the research team by reply–paid envelopes. Data was entered into REDCap electronic data capture tools hosted at CliniKids and audited by a research team member.

## 5. Statistical Analysis

Data handling, Storage, and Confidentiality: The Australian guidelines of the National Health and Medical Research Council (NHMRC) were followed for data handling, storage and protecting confidentiality [36].

Reporting: The CONSORT 2025 checklist was followed when reporting the results [37].

Analysis of Microbial Data: DNA was extracted from 200 mg of faecal material using the DNeasy PowerSoil Pro kit (Qiagen, Hilden, Germany), following the manufacturer’s instructions. The bacterial community composition of faecal samples was assessed using PCR amplification of the V3-V4 region of the 16S rRNA gene with primers 341F and 785R. Library preparation and sequencing was performed by the Ramaciotti Centre for Genomics (UNSW Sydney, Australia), generating 2 × 300 bp paired-end sequence libraries on the NextSeq500 platform (Illumina, San Diego, CA, USA).

The raw data were initially trimmed, quality filtered with TRIMMOMATIC version 0.38 [38], merged, filtered, dereplicated, chimaera-removed, and clustered into amplicon sequence variants (ASVs) using USEARCH v11.0.667 and its UNOISE3 algorithm [39]. Using the UCHIME2 algorithm in USEARCH, the remaining chimeric sequences were detected and removed through reference-based comparison against the GTDB r214 database [40]. The resulting high-quality non-chimeric sequences were taxonomically annotated using the blastn algorithm in BLAST+ 2.7.1 against the GTDB r214 database [40]. To normalise uneven sequencing depths across samples, the total reads of ASVs in each sample were rarefied to the lowest number observed across all samples for subsequent analyses. Microbial community composition was analysed using the *vegan* package [41] in R v4.5.0 (R Core Team, 2025) [42] and Primer v7 (PRIMER-E, UK). Alpha diversity statistics for ASV richness (S), Shannon (*H*′), Gini–Simpson (1-D) and Pielou’s evenness (*J*′) were calculated from the normalised data and compared between sample groups with *t*-tests. Bray–Curtis and Jaccard dissimilarity matrices were constructed using square-root-transformed and presence/absence data, respectively. Non-metric multi-dimensional scaling (nMDS) plots were generated with *ggplot* [43] to visualise multivariate patterns in the community. Permutational multivariate analysis of variance (PERMANOVA) [44] and permutational analysis of multivariate dispersions (PERMDISP) were conducted on paired samples with Primer v7 (PRIMER-E, UK) [44] using type III sums of squares, under 9999 permutations, with fixed factors ‘treatment’ (levels A and B) and ‘timepoint’ (levels pre- and post-), and random factor ‘subject’ (23 levels) nested in ‘treatment’.

To detect changes in the relative abundance of ASV between treatment groups and timepoints, ASVs were fitted to a linear mixed-effects model using the *lme4* package in R. [45] ASV counts of paired samples were converted to relative abundance (0–1), then normalised with the arcsine transformation to stabilise variances. The linear mixed model was designed to have fixed effects of ‘treatment’, ‘timepoint’, and ‘timepoint: treatment’, and the random effect ‘subject’. Analysis of variance (ANOVA) using type III sums of squares was used to test for changes (*p* value cut-off of 0.05) with the package *lmerTest* [46]. Pairwise tests were also conducted between treatment (levels A and B) and timepoint (levels pre- and post-) using the *emmeans* package [47]. To visualise changes in abundance, ASVs were plotted on a heatmap using the package *pheatmap* [48]. Bacterial groups of interest were tested for significant changes in average relative abundance between treatment groups with a *t*-test, and for relative change (equation: (v/vref) −1) in individual children.

Analysis of SCFA and Clinical Data: For the analysis of SCFA, a paired two-sample, two-tailed, *t*-test was used to test for difference within treatment groups, and a two-sample, two-tailed, Welch’s *t*-test was used between the treatment groups. Continuous variables were compared using the two-tailed *t*-test. Categorical variables were compared using Fisher’s exact test. A *p*-value < 0.05 was statistically significant.

## 6. Results

**Figure 1:**
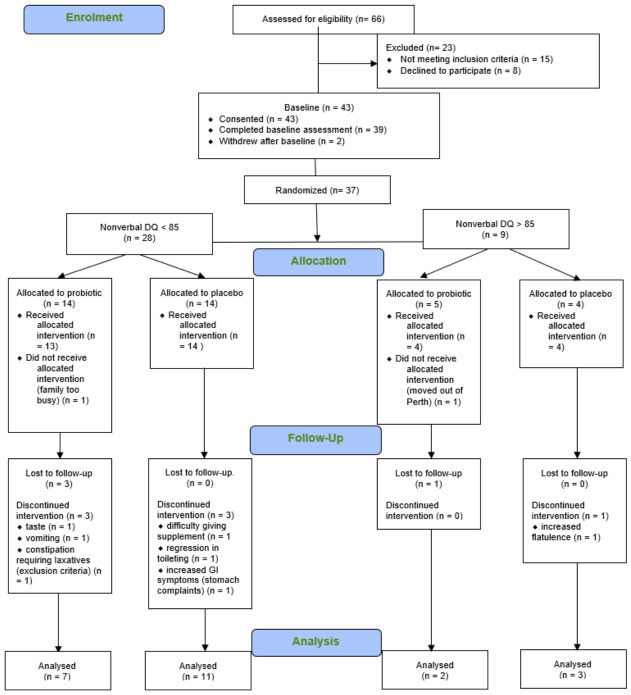
CONSORT Flow Diagram.

For the 66 children screened for eligibility in the trial, consent was received for 43, and 37 were randomly assigned to treatment groups (Figure 1). After excluding withdrawals and loss to follow-up, 23 children with ASD were included for analysis. Faecal samples from these 23 children were obtained at both pre- and post-timepoints [Treatment A (Probiotic): 9; Treatment B (Placebo): 14], referred to as paired samples, and 12 children had only pretreatment faecal samples. There were no significant differences at baseline in any sample characteristics, including the Mullen Scales of Early Learning (MSEL) and the Preschool Language Scale (PLS-5) (Table 1).

Primary Outcome: A total of 1641 ASVs were identified from all microbiota samples (*n* = 46): 1254 ASVs in pretreatment A samples (*n* = 9); 1366 in pretreatment B (*n* = 14); 1258 from post-treatment A (*n* = 9); and 1409 from post-treatment B (*n* = 14). The ASVs comprised nine bacterial phyla, with the majority assigned to Bacillota_A (synonym: Firmicutes_A) (~50%), Bacteroidota (~18%) and Actinobacteriota (~10%). The relative abundances of bacterial classes and microbiota sequencing depth in each sample can be found in the Supplementary Materials (Supplementary Figure S1 and S2).

**Figure 2:**
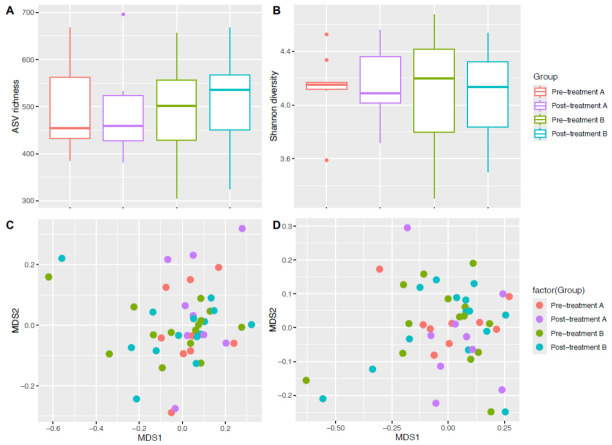
Microbial diversity and community structure and composition of probiotic (treatment A, n = 9) and placebo (treatment B, n = 14) groups. Alpha- and beta-diversity of paired samples comprising four groups: pre-treatment A, post-treatment A, pre-treatment B and post-treatment B. Box plots of (**A**) ASV richness and (**B**) Shannon diversity display the IQR; the line, median; error bars (SD), the range; and the dots represent outliers. The non-metric multi-dimensional scaling (nMDS) plots display the (**C**) square-root-transformed Bray–Curtis dissimilarity and (**D**) the presence/absence of Jaccard dissimilarity among sample groups.

There were no significant differences in alpha-diversity indices between treatment and the placebo groups (Figure 2, Supplementary Table S1). The bacterial communities of each treatment group at pre- and post-timepoints shared an average Bray–Curtis similarity of ~42% and a Jaccard similarity of ~40%, and no visual clustering of community structure or composition by treatment group or timepoint weas observed by non-metric multi-dimensional scaling (Figure 2). PERMANOVA also found no significant differences in bacterial community structure and composition in any comparisons between or within treatments and timepoints (*p* > 0.05) (Supplementary Table S2). As individual children were re-sampled (pre- and post-), the factor ‘subject’ was included in the PERMANOVA model which showed significant variation in the microbial community between children (*p* = 0.0001).

**Figure 3:**
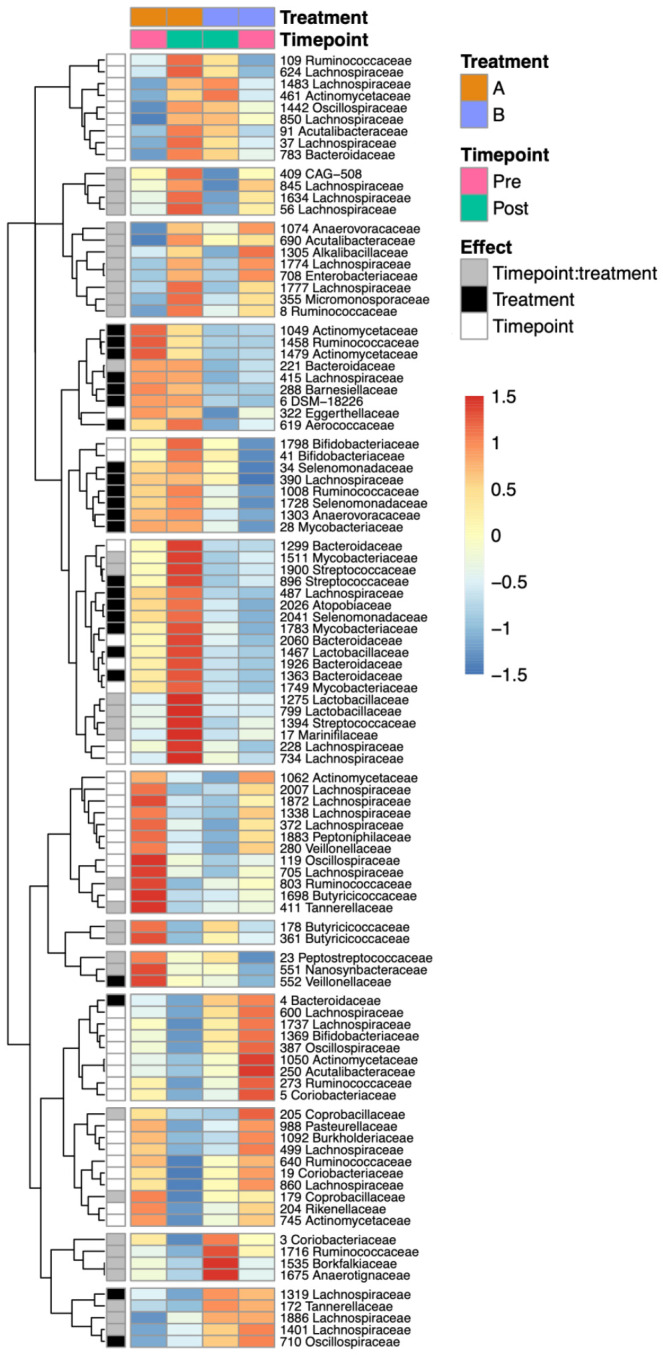
Heatmap of amplicon sequence variants (ASVs) with identified changes in abundances between probiotic (treatment A) and placebo (treatment B) groups and timepoints. ASV names are numerical identifiers followed by family name. Averaged ASV counts for pre-treatment A, post-treatment A, pre-treatment B and post-treatment B are log10-transformed (+1), scaled by row, and clustered by average method. Row annotation ‘effect’ reflects which fixed-effects changes were identified in with ANOVA. The scale displays the z-score mean as 0 and standard deviations as 1.

**Figure 4:**
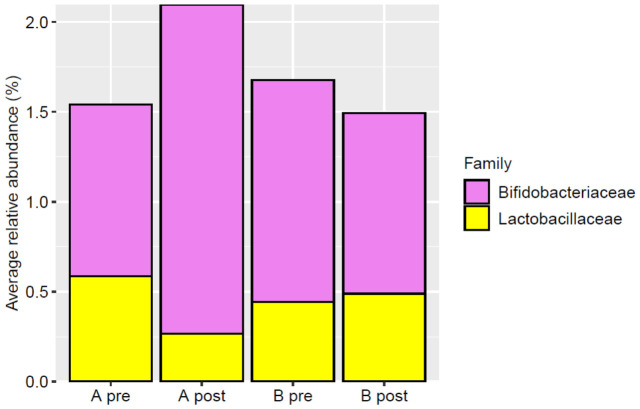
Average relative abundance (%) of Bifidobacteriaceae and Lactobacillaceae in probiotic (treatment A) and placebo (treatment B) groups at pre- and post-treatment timepoints. There was a significant increase in Bifidobacteriaceae in post-treatment A samples, determined by a paired two-sample, one-tail *t*-test (*p* = 0.047).

While overall microbial community as assessed by similarity indices did not differ between treatment groups and timepoints, the linear mixed-effects model analysis on individual ASVs identified 102 with changes in abundance (*p* < 0.05) (Figure 3). Of these, 34 changed with the factor ‘timepoint: treatment’, 44 with ‘timepoint’ only, and 24 with ‘treatment’ only. The most represented families were Lachnospiraceae (29 ASVs), followed by Ruminococcaceae (8) and Bacteroidaceae (7). As strains belonging to Bifidobacteriaceae and Lactobacillaceae make up Vivomixx^®^, the relative abundance of these two families was examined. The average relative abundance of Bifidobacteriaceae significantly increased post-treatment in the probiotic group (*p* = 0.046), which was not observed post-treatment in the placebo group (Figure 4). There were no significant increases in Lactobacillaceae in either treatment group. As individual variability was determined to be a significant factor, relative changes in each child were also examined. In the probiotic treatment group, six of the nine children displayed an increase in Bifidobacteriaceae (group average 154.4), four of which were greater than the average relative change in the placebo group (group average 1.39) (Supplementary Table S3). The same pattern was not observed for Lactobacillaceae.

**Figure 5:**
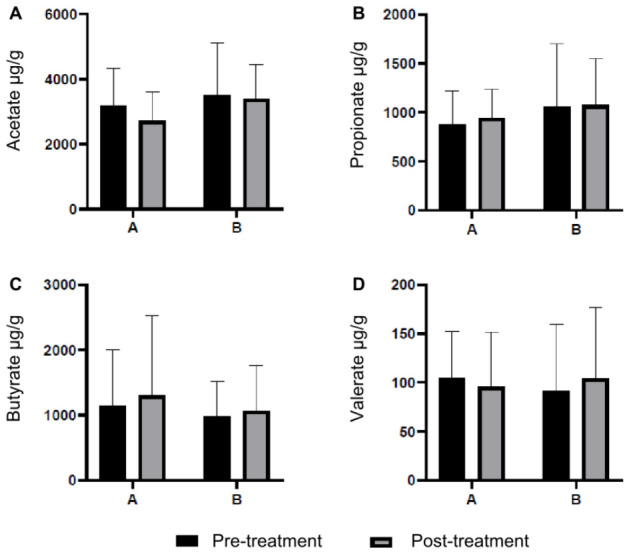
Average concentration (µg/g wet faeces) of (**A**) acetate, (**B**) propionate, (**C**) butyrate and (**D**) valerate in probiotic (treatment A) and placebo (treatment B) group faecal samples at pre- and post-treatment timepoints. Short-chain fatty acids were quantified using gas chromatography with a flame ionisation detector. No significant differences were detected between treatments or timepoints (*p* ≥ 0.05). Error bars display the standard deviation.

Secondary Outcomes: There were no significant differences between the probiotic (treatment A) and placebo group (treatment B) at baseline or post-intervention in any of the four parent-reported behavioural questionnaires (Supplementary Table S4). The SCFA analysis found no differences in the concentration of faecal acetate, propionate, butyrate or valerate between treatment groups and timepoints (Figure 5), or patterns of relative change (Supplementary Tables S5 and S6). AES was completed by 17 participants (Probiotics: 6 and Placebo: 11). The AFRS scores were comparable between groups at both time points (Supplementary Table S4).

## 7. Discussion

This pilot RCT examined the effect of multi-strain probiotic supplementation on the gut microbiota (primary outcome) in young children with ASD. Overall, we found no significant overall changes in gut microbiota between the probiotic and placebo group. However, there was a significant increase in faecal Bifidobacteriaceae in children supplemented with the probiotic compared with placebo. The negative findings of our study may relate to the relatively short intervention period, dietary heterogeneity, or the substantial variability of the baseline microbiome. However, the supplementation duration of 12 weeks is adequate for expecting probiotics’ effects, based on the knowledge available in this field. Previous studies have reported reduced faecal bifidobacteria in children with ASD, which may contribute to altered neurotransmitter levels [49,50]. Therefore, an increase in faecal bifidobacteria may have the potential to improve behaviour among children with ASD [51]. An extended discussion on the microbiota and SCFA findings are provided in the Supplementary Materials. Behavioural (secondary) outcomes showed no significant changes, likely due to the small sample size as well as the trial protocol and formulation. No significant adverse effects were noted in trial participants. Loss to follow-up was significant, with recruitment of only 23 rather than the expected 40 participants.

The challenges encountered in recruitment and retainment during the trial need to be discussed. Out of 66 parents and children assessed for eligibility, 8 declined to participate for reasons that were not declared. However, a range of factors may explain their decision [52]. Parents are known to face significant stress due to the high level of care required for a child with ASD and the added financial burden [52]. Coping with the logistics of trial participation (e.g., hospital visits, the collection and storage of faecal samples at home, and assuring their timely collection by the courier) may have been difficult for parents. The consequences of the additional stress of taking a child with ASD for frequent and long sessions of multiple therapies outside the trial while handling daily family responsibilities is significant in this context.

During the later stages of the trial, several participants discontinued the intervention, primarily due to symptoms such as vomiting, constipation, difficulty in administering supplements (“did not like the taste”), regression in toileting skills, and increased flatulence in the child. It is important to note that these symptoms were equally reported in both probiotic and placebo groups. Most of these minor symptoms resolved after cessation of supplement. Gastrointestinal disorders are among the common coexisting morbidities in children with ASD. Studies have reported a correlation between argumentative, oppositional defiant and destructive behaviours and gastrointestinal symptoms in this cohort. Challenging and unexplained behaviours have been attributed to a child’s inability to communicate discomfort in response to gastrointestinal distress [53]. Hence, it is possible that minor symptoms or changes in the child’s behaviour prompted parents, who were already under stress, to discontinue the trial supplementation. Our findings suggest the need for ongoing psychological support for parents participating in such trials. The other important factor, which possibly impacted recruitment significantly, was COVID-19. The widespread disruption caused by the pandemic affected both recruitment and staffing. The travel restrictions during COVID-19 and heightened concerns about safety created a substantial barrier to enrolment [54].

There is conflicting evidence regarding the impact of probiotic supplementation on the gut microbiota and symptoms of ASD, which may be related to heterogeneity in methodology, including the selected probiotic strain/s, duration of supplementation, behavioural outcome measures, and sample sizes [55]. Variations in baseline gut microbiota may also explain the differences in the outcomes of probiotic trials in children with autism, as we observed in our study, with individual children displaying significant variation. Dysbiosis, characterised by increased abundance of pathogens and lower abundances of *Bifidobacterium* and *Prevotella*, is reported in children with ASD [50,56]. A recent RCT [57] enrolled 80 children (autism: 41; ADHD: 39; age: 5–14 years) supplemented with *L. plantarum* CECT7485 (KABP023) and *L. brevis* CECT7480 (KABP052) at a dose of 1 × 10^9^ CFU. Among the children with autism, probiotic supplementation was associated with increased diversity and abundance of *Bacteroides*, *Bacilli*, and *Actinobacteria*, and decreased abundance of bacteria linked to gastrointestinal and behavioural problems (e.g., *Eggerthellaceae*). The investigators concluded that the variation in gut microbiota influences probiotic effects [57]. In turn, variation in gut microbiota is associated with factors such as dietary habits, lifestyle, genetics, and environmental influences [58].

Considering the significance of product and strain heterogeneity, it is important to consider the results of previous studies on Visbiome^®^, the probiotic used in our study. Billeci et al. [59]) assessed the effect of Visbiome^®^ on brain function in 46 children (mean age 46.56 months ± 13.92) with ASD using an electroencephalogram (EEG). The results of their 6-month RCT showed decreased power in the frontopolar regions in beta and gamma bands, and increased coherence in the same bands, together with a shift in frontal asymmetry, suggesting a modification toward typical brain activity. EEG measures were significantly correlated with clinical and biochemical measures [59]. Santocchi et al. [60] evaluated a probiotic’s (Visbiome^®^) effects on brain activity and function in 85 pre-school children with ASD (mean age, 4.2 years; 84% boys) in an RCT. Total Autism Diagnostic Observation Schedule–Calibrated Severity Score (ADOS-CSS), the primary outcome, was not significantly different between groups. An exploratory analysis in a subgroup of children showed significant improvement in gastrointestinal symptoms, adaptive functioning, and sensory profiles in the gastrointestinal group children treated with the probiotic compared with placebo [60].

In another small (n = 10) RCT, Arnold et al. (2019) [61] examined the effects of eight weeks’ supplementation with Visbiome^®^ on children (aged 3 to 12 years) with ASD using the paediatric quality of life inventory (PedsQL) GI module, parent-rated anxiety scale for ASD (PRAS-ASD) and microbial community analysis. They reported that each measured outcome improved from baseline, with the probiotic group (n = 6) showing more improvement than placebo (n = 4); however, these improvements were not statistically significant. They were also unable to detect differences in the overall microbial community between treatment groups. One interesting observation was that PedsQL measures significantly correlated with the abundance of *Lactobacillus*. Unlike our study, they observed no significant changes the abundance of bacterial families (e.g., Bifidobacteriaceae) post-probiotic or placebo treatments. However, GI complaints showed a significant improvement in the probiotic group compared to placebo. Many of these findings align with our trial, except for the apparent inversion in the significance of Bifidobacteriaceae and Lactobacillaceae. Arnold et al. also reported heterogeneity between the microbiota of individual children, resulting in dynamic changes unique to each child, similar to what we observed in our study. This observation was also made by Novau-Ferre et al. (2025), with the authors concluding that variation in gut microbiota influences probiotic effects [57]. Indeed, it has been demonstrated by Zmora et al. (2018) that humans display individual-specific gut mucosal colonisation resistance, and the universal effectiveness of probiotic supplementation may therefore be limited, meriting the development of personalised probiotic approaches [62].

## 8. Conclusions

The robust design of this study was undermined by challenges with participant recruitment and retention, exacerbated by the COVID pandemic. Addressing the reasons for the sinificant loss to follow-up is critical for designing and conducting adequately powered RCTs in this field. Nevertheless, we detected a significant increase in Bifodobacteriaceae after supplementation with the probiotic compared to the placebo. It is possible that children experiencing more severe gastrointestinal (GIT) or behavioural symptoms were more likely to participate in, as well as drop out of, our study. Further RCT can address the issue of selection bias by recruitment in the context of the severity of illness. Evaluating the clinical significance of this increase should be considered in future trials.

## Figures and Tables

**Table 1:** Baseline Characteristics of Participants.

	Probiotic *n* = 9 Mean (SD)	Placebo *n* = 14 Mean (SD)	*p* Value
Child age, years	4.10 (0.72)	3.99 (0.75)	*p* = 0.73
Gender (male, %)	7 (77.8%)	11 (78.6%)	*p* = 0.96
SEIFA, IRSAD n (%) > 4	7 (77.8%)	12 (85.7%)	*p* = 0.62
MSEL NVDQ	65.94 (32.82)	61.26 (25.38)	*p* = 0.70
PLS-5, Total	76.33 (31.01)	63.60 (14.54)	*p* = 0.28

SEIFA—Socio-Economic Indexes for Areas; IRSAD—Index of Relative Socio-Economic Advantage and Disadvantage; MSEL—Mullen Scales of Early Learning; NVDQ—Nonverbal Developmental Quotient; PLS-5—Preschool Language Scale-5; averaged values are presented ± standard deviation.

## Data Availability

The data presented in this study are available on request from corresponding author subject to clearance from hospital ethics committee.

## Supplementary Materials

The following supporting information can be downloaded at: https://www.mdpi.com/article/10.3390/nu18132079/s1, Figure S1: Microbiota sequencing depth. Rarefaction curve displaying satisfactory sequencing depth as ASV count curves tend towards the horizontal; Figure S2. Relative abundance (%) of bacterial classes in (A) pre-treatment and (B) post-treatment faecal samples; Table S1: Comparison of alpha diversity measures between probiotic (treatment A) and placebo (treatment B) groups; Table S2. Results summary of PERMANOVA and PERMDISP on ASV microbial community structure and composition of faecal samples, based on square-root-transformed Bray–Curtis (abundance) and Jaccard (presence-absence) similarity matrices; Table S3. Percentage relative abundance of Bifidobacteriaceae and Lactobacillaceae at pre- and post-treatment timepoints for each child; Table S4. Parent-reported behavioural assessments of subjects in probiotic (treatment A) (n = 9) and placebo (treatment B) (n = 14) groups at pre- and post-time points; Table S5. Concentration of acetate and butyrate (µg/g wet faeces) at pre- and post-treatment timepoints for each child; Table S6. Concentration of propionate and valerate (µg/g wet faeces) at pre- and post-treatment timepoints for each child [63,64,65,66,67,68,69,70,71,72,73,74,75,76,77,78,79,80,81,82,83,84,85,86,87,88,89,90].
