# Supplementary material for: Effect of Probiotic Supplementation on Gut Microbiota in Children with Autism: A Pilot Randomised Controlled Trial
Source: Nutrients. 2026 Jun 25;18(13):2079. doi: 10.3390/nu18132079 (PMC13363267; doi:10.3390/nu18132079)
Supplement: Supplementary file 1 [file nutrients-18-02079-s001.zip › nutrients-4335313-supplementary.pdf]

Supplementary information: Effect of Probiotic Supplementation on Gut Microbiota in Children with Autism: A Pilot Randomised Controlled Trial.

Sachin Agrawal, Shripada Rao, Andrew Whitehouse, Gail A Alvares, Alpana Kulkarni, Jessica A. Taylor, Patricia L. Conway, Torsten Thomas and Sanjay Patole.

Methods

Sample Size Estimation: Based on the simulated gamma parameter of the Dirichlet-multinomial distribution [63] (R package 'HMP') in the autism metagenomics data [64]. The frequency profile of the top 14 most abundant genera (cumulative frequency >95% of the total quantifiable reads) from 40 individuals with autism and 31 neurotypical individuals in public data was used as input. A sample size of 20 children per group (50,000 16S reads; ~50 million shotgun reads) was estimated to have ~80% power to detect the compositional taxonomic difference in the gut microbiome at a 5% α level. The power of detecting the compositional difference between two groups (autism and healthy control) at a given level of type 1 error (5%) depends on two factors: the sample size and the number of reads. The read number of 50,000 is one of the common throughputs for 16S rRNA gene sequencing (corresponding to ~5 GB of shotgun metagenomic sequencing). We used this fixed number of reads in our power evaluation. The number of samples in each group influences the type 2 error (and power) in detecting compositional differences between two groups, as determined by metagenomic simulations. The number of Monte Carlo experiments was set to 5,000 for each simulated sample. The approach to analyses was based on the principle of intention-to-treat.

Selection of Probiotics: Our probiotic selection and protocol is based on ongoing *(ClinicalTrials.gov Identifier: NCT03369431, NCT02708901, NCT02903030)* and previous studies [65-68], including a survey reporting that multi-strain high-dose probiotics may be more effective in conditions associated with severe and chronic gut dysbiosis [69], and the recommendations of an expert group for probiotic RCTs in ASD.

Stool Sample Collection: Stool samples were collected as soon as possible after enrolment, but before commencing the trial supplement, and within one week after completing the 4 months of supplementation. If any child dropped out of the study before four months, every effort was made to collect the stool samples at that stage. Written instructions were provided to parents to collect the stool samples in the yellow specimen jars supplied at enrolment. Parents were asked to keep the collected sample in their kitchen fridge at 4-6 °C (not the freezer compartment) and to call the study coordinator. An accredited courier was sent to the patient's home to collect the sample and bring it back to CliniKids, where the samples were stored at -80 °C in cryovials. Stool sample analysis was conducted for faecal microbiota and short-chain fatty acids [70-73].

Analysis of Faecal Short-Chain Fatty Acids (SCFA): Volatile SCFAs were quantified using gas chromatography (GC-2010, Shimadzu Corporation, Kyoto, Japan) with a flame ionization detector (FID) at the Singapore Centre for Environmental Life Sciences Engineering (SCELSE). Briefly, 200 mg of faecal material were suspended in 1 mL of 1% phosphoric acid solution and homogenized via vortex for 20 min. Next, the samples were centrifuged at 20,850 × g for 20 min, and the supernatant was transferred into a new tube. Then, the supernatant was sterile-filtered through a 0.22 μm spin column filter and diluted tenfold in sterile Milli-Q water. Diluted filtrate (900 μL) was transferred into a glass autosampler vial, and 100 μL of 1% phosphoric acid was added to the vial for GC-FID analysis with column DB-FFAP (30 m, 0.25 mm, 0.25 μm) for gas separation. The operating conditions were as follows: inlet temperature: 250°C; injection mode: split; column oven temperature program: 100°C hold 5 min, rate 20 to reach 150°C and zero hold time, rate 5 to reach 200°C and zero hold time, rate 20 to reach 240°C and hold 2 mins (total program time: 21.50 mins); FID temperature: 300°C; composition gas: N2; composition flow: 30ml/min; H2 flow: 40ml/min; air flow: 400 ml/min. Standards of acetate, propionate, butyrate, and valerate were serially diluted with sterile Milli-Q water to achieve known individual standard concentrations of 200 ppm, 100 ppm, 50 ppm, 25 ppm, 5 ppm, and 1 ppm. The standards were also transferred into glass autosampler vials by adding 900 uL of the prepared standard and 100 uL of 1% phosphoric acid. Concentrations of acetate, propionate, butyrate, and valerate were calculated using linear standard curves (1 to 200 ppt) and expressed as μg/g of wet faeces.

Developmental assessments:

(a) **The Short Sensory Profile (SSP-2): The SSP is a caregiver-report questionnaire applicable to** children aged 3–14 [74]. The 34-item questionnaire employs a 5-point Likert scale for caregivers to report the frequency with which their child responds to sensory input during daily activities. Internal consistency (0.79–0.86), test–retest stability (0.83–0.97) and inter-rater reliability (0.70–0.80) are all adequate.

(b)**Repetitive Behaviour Scale – Revised (RBS-R):** The RBS-R is a 43-item caregiver-report questionnaire. Each item is scored on a four-point Likert scale ranging from 0 (behaviour does not occur) to 3 (behaviour occurs and is a severe problem) [75]. Higher scores indicate greater levels of atypicalities.

(c)Vineland Adaptive Behavioural Scales—2nd edition (VABS-III): The VABS III is a commonly used measure of adaptive functioning in the domains of communication, social skills, daily living skills, and motor skills for individuals aged from birth through 90 years of age. The current study will utilise the caregiver-completed 'survey form' of **VABS-III**, which takes ~20 minutes to complete [76].

(d) Mullen's Scale of Early Learning (MSEL): This is a standardised developmental assessment of the cognitive functioning of young children from birth to 68 months. It is based on the child's responses to activities prepared by the examiner [31]. It takes ~40 minutes to complete and measures five skill domains; however, for this study, only the fine motor and visual reception subscales will be undertaken to calculate a "learning nonverbal developmental quotient composite score". Scores from each of these scales are transformed into T-Scale scores, centred around a mean of 50 (SD=10). Scores from the subtests are combined to form an 'Early Learning Composite', which provides a measure of overall neurocognitive development (M = 100, SD = 15). Test–retest reliability is adequate (M=.90; R=.71–.96) and inter-scorer reliability is strong (R=.91–.99).

(e) Social Responsiveness Scales (SRS-2): A parent-completed questionnaire of social competence from 5-month to 18-year-olds. It comprises 65 items using a '0' (not true) to '4' (almost always true) point Likert scale. The questions focus on the child's behaviour over the past 6 months. Internal consistency is high for male and female participants (Cronbach's α>0.90). The instrument has temporal stability (test–retest reliability at 17 months: r=0.85 for males and 0.77 for females). Inter-rater reliability is high between mothers and fathers (0.91). The SRS discriminates well between children with ASD and other psychiatric conditions [77].

Dietary Changes: The Australian Child and Adolescent Eating Survey (AES): The AES is used to measure the nutritional intakes of children and adolescents aged 2-17 years. It is a validated and reliable tool to measure usual food and nutrient intake over the past 3-6 months in this population [78],[79]). The AES is a 120-item semi-quantitative food frequency questionnaire (FFQ) that is self-reported or completed by parents regarding the frequency of food consumption. Frequency options for most items range from 'Never' to '4 or more times per day' for food items and '7 or more glasses per day' for beverages but vary depending on the item. Nineteen questions relate to the intake of vegetables and 11 items to fruit, with a separate section for seasonal fruit. The completed AES FFQ generates individual mean daily macro- and micronutrient intake and reports on the proportion of healthy and unhealthy food habits. It takes less than 20 minutes to complete. The survey will be administered at baseline and after the completion of the study period (4 months) (*https://australianeatingsurvey.com.au/).*

Rationale for AES: Children with autism spectrum disorder (ASD) are often described as picky eaters with low intake of fibre-rich foods, including fruits and vegetables. Dietary patterns are known to be related to faecal microbiota composition in children with ASD [80]. Hence, diet can be a confounding factor for faecal microbiota composition in children with ASD. The results of the AES will be helpful in the robust analysis of faecal microbiota in children participating in our trial of probiotic supplementation.

Results

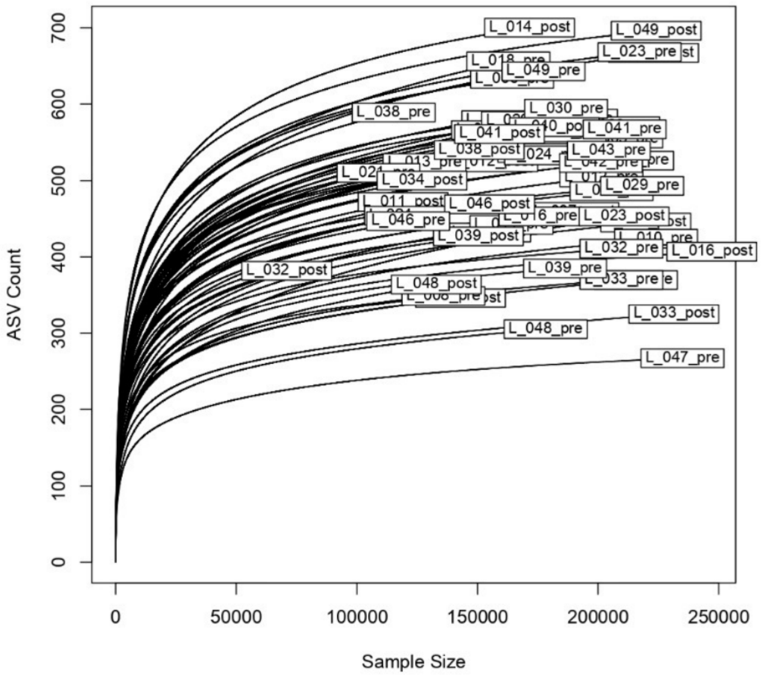

Figure S1. Microbiota sequencing depth. Rarefaction curve displaying satisfactory sequencing depth as ASV count curves tend towards the horizontal.

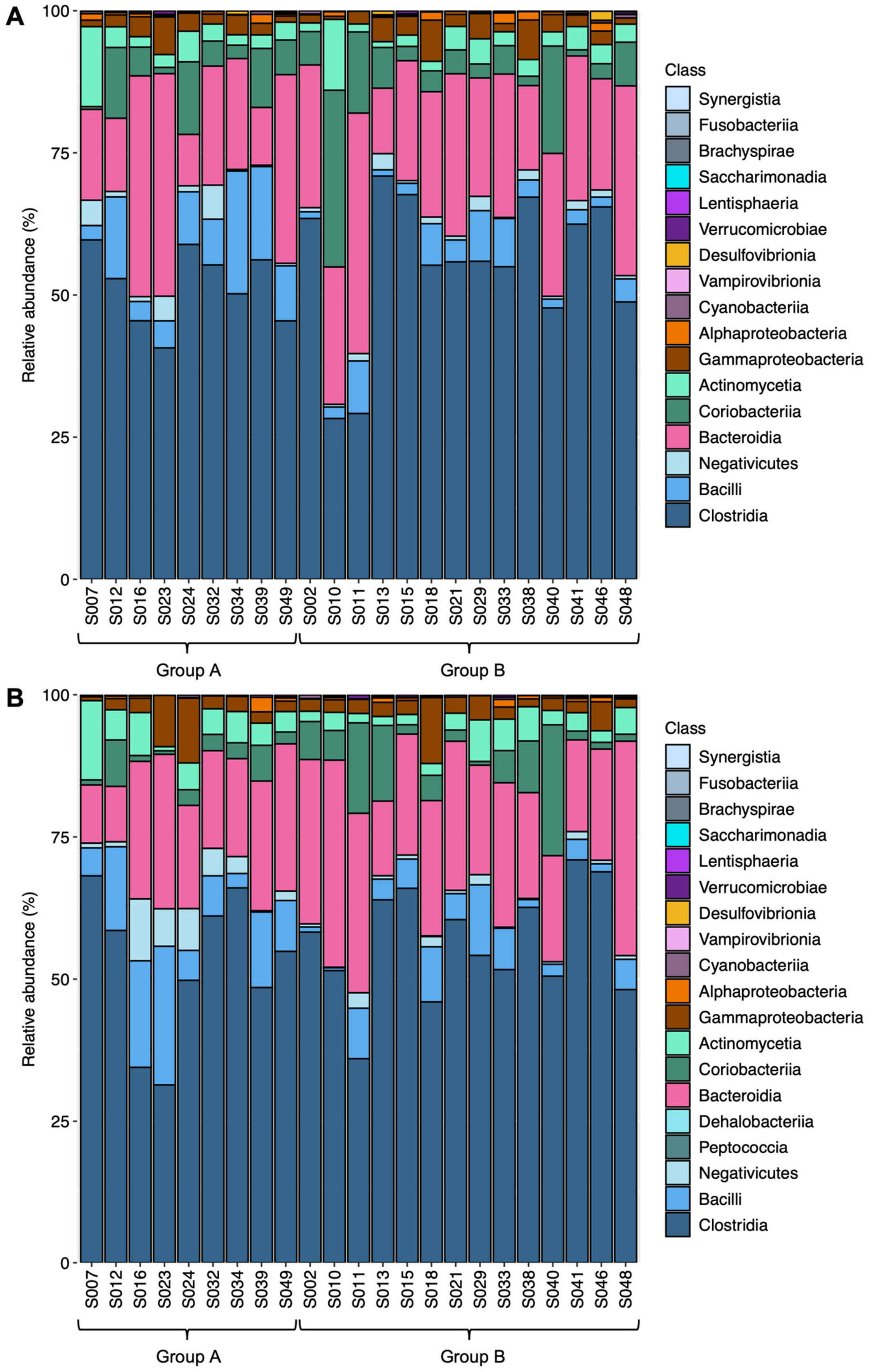

Figure S2. Relative abundance (%) of bacterial classes in (**A**) pre-treatment and (**B**) post-treatment faecal samples from individual children divided into two treatment groups: probiotic (group A) and placebo (group B). Taxonomy was assigned to amplicon sequence variants using the Genome Taxonomy Database (release 214).

Table S1. Comparison of alpha diversity measures between probiotic (treatment A) and placebo (treatment B) groups. Average number of ASVs (richness), minimum number of ASVs in a sample (min.), maximum number of ASVs in a sample (max.), Shannon's (H') and Gini–Simpson's (1-D) diversity indices and Pielou's evenness (J') of paired samples comprising four groups: pre-treatment A (n = 9), post-treatment A (n = 9), pre-treatment B (n = 14) and post-treatment B (n = 14). A paired two-sample, two-tailed t-test was used to test for differences within treatment groups, and a two-sample, two-tailed Welch's t-test was used between treatment groups. Averaged values are presented with ± standard deviation.

**Group**	**Richness (S)**	**min.**	**max.**	**Shannon (H')**	**Simpson (1-D)**	**Evenness (J')**
Pre-treatment A	501.44 ±101.7	385	668	4.144 ±0.249	0.965 ±0.010	0.668 ±0.027
Post-treatment A	486.78 ±93.74	382	696	4.127 ±0.276	0.967 ±0.012	0.663 ±0.031
Pre-treatment B	484.86 ±97.28	305	656	4.105 ±0.433	0.958 ±0.027	0.665 ±0.054
Post-treatment B	499.14 ±100.2	325	667	4.072 ±0.344	0.960 ±0.018	0.657 ±0.041
**T-test *p* value**						
Pre- vs. post-treatment A	0.634			0.883	0.806	0.725
Pre- vs. post-treatment B	0.172			0.548	0.767	0.303
Pre-treatment A vs. B	0.703			0.788	0.398	0.855
Post-treatment A vs. B	0.767			0.678	0.268	0.710

Table S2. Results summary of PERMANOVA and PERMDISP on ASV microbial community structure and composition of faecal samples, based on square-root-transformed Bray–Curtis (abundance) and Jaccard (presence-absence) similarity matrices.

**Abundance PERMANOVA main test**						
**Source**	**df**	**SS**	**MS**	**Pseudo-F**	**U. Perms**	***p* (perm)**
Timepoint	1	730.5	730.5	1.2660	9875	0.1414
Treatment	1	2214.0	2214.0	0.7928	9786	0.8864
Treatment x timepoint	1	561.1	561.1	0.9723	9889	0.4975
Subject (treatment)	21	58643.0	2792.5	4.8394	9626	0.0001
Residuals	21	12118.0	577.0			
Total	45	74220.0				
**Abundance PERMANOVA pairwise tests**						
**Treatment x timepoint**				***t***	**U. Perms**	***p* (perm)**
**Within timepoint level pre:**						
A vs B				0.7964	9784	0.9965
**Within timepoint level post:**						
A vs B				1.0054	9791	0.4157
**Within treatment level A:**						
Pre vs post				0.9916	9912	0.5002
**Within treatment level B:**						
Pre vs post				1.0416	9917	0.3519
**Abundance PERMDISP**						
**Factor**			**df1**	**df2**	**F**	***p* (perm)**
Timepoint			1	44	0.0555	0.8361
Treatment			1	44	0.0172	0.9038
**Presence–absence PERMANOVA main test**						
**Source**	**df**	**SS**	**MS**	**Pseudo-F**	**U. Perms**	***p* (perm)**
Timepoint	1	760.0	760.0	1.1450	9884	0.2282
Treatment	1	2389.6	2389.6	0.7899	9807	0.9255
Treatment x timepoint	1	673.3	673.3	1.0143	9853	0.4225
Subject (treatment)	21	63532.0	3025.3	4.5578	9628	0.0001
Residuals	21	13939.0	663.8			
Total	45	81257.0				
**Presence–absence PERMANOVA pairwise tests**						
**Treatment x timepoint**				***t***	**U. Perms**	***p* (perm)**
**Within timepoint level pre:**						
A vs B				0.8550	9756	0.9950
**Within timepoint level post:**						
A vs B				0.9633	9750	0.6100
**Within treatment level A:**						
Pre vs post				1.0292	9897	0.4092
**Within treatment level B:**						
Pre vs post				0.9969	9895	0.4555
**Presence–absence PERMDISP**						
**Factor**			**df1**	**df2**	**F**	***p* (perm)**
Timepoint			1	44	0.1104	0.7621
Treatment			1	44	0.7782	0.4405

Table S3. Percentage relative abundance of Bifidobacteriaceae and Lactobacillaceae at pre- and post-treatment timepoints for each child. Relative change ((v/vref) -1) values reflect an increase (positive) or decrease (negative) in relative abundance post-treatment. Average relative abundances for treatment groups A (probiotic) and B (placebo) are presented ±standard deviation. Note that participant ID is random and cannot be used to identify an individual child.

	**Bifidobacteriaceae**			**Lactobacillaceae**		
**Participant ID**	**Pre**	**Post**	**Relative change**	**Pre**	**Post**	**Relative change**
S007	5.465	8.389	0.53	0.211	0.263	0.25
S012	0.746	2.313	2.10	0.018	0.076	3.14
S016	0.274	0.020	-0.93	0.158	0.159	0.00
S023	0.029	0.011	-0.63	0.094	0.039	-0.58
S024	1.417	0.292	-0.79	1.824	0.213	-0.88
S032	0.320	0.609	0.91	0.163	0.460	1.83
S034	0.294	1.134	2.86	0.999	0.004	-1.00
S039	0.042	2.956	68.71	1.795	1.056	-0.41
S049	0.001	0.743	1316.84	0.014	0.125	8.20
**A group average**	0.95 ±1.75	1.83 ±2.66	154.40	0.59 ±0.76	0.27 ±0.33	1.17
S002	0.341	0.834	1.44	0.026	0.402	14.22
S010	11.120	2.356	-0.79	0.563	0.050	-0.91
S011	0.150	0.590	2.93	0.033	0.086	1.63
S013	0.104	0.084	-0.19	0.237	0.243	0.03
S015	0.017	0.037	1.23	0.043	0.144	2.31
S018	0.224	0.129	-0.42	0.203	0.002	-0.99
S021	1.387	0.160	-0.88	0.001	0.005	3.92
S029	0.271	4.176	14.40	0.003	0.861	312.12
S033	0.211	0.078	-0.63	3.859	3.465	-0.10
S038	0.920	3.774	3.10	0.215	0.333	0.55
S040	0.719	0.164	-0.77	0.079	0.047	-0.40
S041	0.036	0.030	-0.18	0.026	0.102	2.93
S046	0.690	1.214	0.76	0.794	0.604	-0.24
S048	1.086	0.435	-0.60	0.120	0.502	3.18
**B group average**	1.23 ±2.88	1.00 ±1.41	1.39	0.44 ±1.01	0.49 ±0.89	24.16

Table S4. Parent-reported behavioural assessments of subjects in probiotic (treatment A) (n = 9) and placebo (treatment B) (n = 14) groups at pre- and post- time points. Continuous variables were compared using the two-tailed t-test and categorical variables were compared using Fisher's exact test. Averaged values are presented ± standard deviation. Note that 6 and 11 subjects from treatment A and treatment B groups, respectively, participated in the Australian eating survey. SSP-2 = Short Sensory Profile.

	**Treatment A**		**Treatment B**		
	**Pre**	**Post**	**Pre**	**Post**	***p* value**
Adaptive Behaviour Scale	64.9 ±8.7	67.1 ±12.1	64.9 ±8.7	69.1 ±9.0	0.91
Social Responsiveness Scale	82.6 ±10.0	79.4 ±10.2	75.3 ±9.6	75.6 ±6.9	0.37
SSP-2: Sensory	40.5 ±16.2	35.9 ±8.9	33.9 ±11.4	34.6 ±10.8	0.32
SSP-2: Behavioural	63.0 ±14.3	59.3 ±15.4	59.3 ±11.8	58.3 ±11.7	0.69
Repetitive Behaviour Scale	35.3 ±17.6	32.9 ±18.5	20.1 ±11.8	19.6 ±8.3	0.67
Australian Eating Survey	28.3 ±7.1	22.3 ±13.0	26.3 ±12.5	27.9 ±12.6	0.07

Table S5. Concentration of acetate and butyrate (µg/g wet faeces) at pre- and post-treatment timepoints for each child. Relative change ((v/vref) -1) values reflect an increase (positive) or decrease (negative) post-treatment. Average concentrations for treatment groups A (probiotic) and B (placebo) are presented ±standard deviation. Note that participant ID is random and cannot be used to identify an individual child.

	Acetate			Butyrate		
**Participant ID**	**Pre**	**Post**	**Relative change**	**Pre**	**Post**	**Relative change**
S007	1069.6	1565.6	0.46	314.6	1036.3	2.29
S012	4851.2	4436.1	-0.09	2993.2	2123.2	-0.29
S016	2213.5	2966.3	0.34	315.3	1026.3	2.26
S023	3602.7	2617.7	-0.27	978.0	316.1	-0.68
S024	4571.8	1812.5	-0.60	1825.9	532.3	-0.71
S032	3311.0	3699.9	0.12	696.8	4264.3	5.12
S034	3296.2	2758.7	-0.16	1496.9	1088.9	-0.27
S039	3266.0	2242.8	-0.31	985.8	389.9	-0.60
S049	2495.1	2261.0	-0.09	619.1	880.0	0.42
**A group average**	3186 ±1162	2706 ±906	-0.07	1136 ±860	1295 ±1236	0.84
S002	2974.6	2671.4	-0.10	1116.3	1002.0	-0.10
S010	1564.1	2278.8	0.46	240.7	370.0	0.54
S011	3796.9	5129.6	0.35	1715.6	1947.6	0.14
S013	7529.7	1944.0	-0.74	1678.9	269.6	-0.84
S015	3671.9	3184.4	-0.13	1046.1	762.2	-0.27
S018	4896.2	4600.2	-0.06	1235.9	2179.0	0.76
S021	3975.4	4632.3	0.17	1814.3	1977.6	0.09
S029	3823.6	3680.5	-0.04	838.4	1814.0	1.16
S033	3165.4	2726.4	-0.14	981.8	302.0	-0.69
S038	3927.4	3898.9	-0.01	756.0	1259.4	0.67
S040	4582.8	4272.6	-0.07	1077.3	1256.7	0.17
S041	1864.1	3619.2	0.94	773.0	898.0	0.16
S046	1699.5	3208.2	0.89	342.3	668.5	0.95
S048	1556.3	1637.7	0.05	103.6	162.3	0.57
**B group average**	3502 ±1613	3391 ±1061	0.11	980 ±530	1062 ±695	0.24

Table S6. Concentration of propionate and valerate (µg/g wet faeces) at pre- and post-treatment timepoints for each child. Relative change ((v/vref) -1) values reflect an increase (positive) or decrease (negative) post-treatment. Average concentrations for treatment groups A (probiotic) and B (placebo) are presented ±standard deviation. Note that participant ID is random and cannot be used to identify an individual child.

	Propionate			Valerate		
**Participant ID**	**Pre**	**Post**	**Relative change**	**Pre**	**Post**	**Relative change**
S007	510.9	540.5	0.06	66.3	161.9	1.44
S012	1040.8	834.4	-0.20	101.8	89.1	-0.12
S016	850.0	979.9	0.15	111.9	7.6	-0.93
S023	999.0	1500.6	0.50	133.9	25.6	-0.81
S024	672.0	660.5	-0.02	79.8	57.3	-0.28
S032	755.7	1094.2	0.45	156.6	142.4	-0.09
S034	1571.8	1232.1	-0.22	188.4	149.4	-0.21
S039	433.5	789.3	0.82	48.8	94.9	0.95
S049	1048.7	845.8	-0.19	55.4	130.5	1.36
**A group average**	875 ±343	942 ±297	0.15	105 ±48	95 ±56	0.14
S002	709.2	791.8	0.12	22.2	18.5	-0.17
S010	351.5	651.3	0.85	34.2	54.0	0.58
S011	2412.4	2113.8	-0.12	31.3	130.0	3.15
S013	2384.5	1027.9	-0.57	237.5	91.2	-0.62
S015	874.8	781.0	-0.11	178.5	90.1	-0.50
S018	1164.9	1795.4	0.54	69.0	126.7	0.84
S021	1150.6	1492.3	0.30	155.2	219.0	0.41
S029	1384.5	1572.4	0.14	158.0	260.1	0.65
S033	1072.5	897.8	-0.16	26.9	8.3	-0.69
S038	948.4	935.5	-0.01	83.2	146.3	0.76
S040	663.6	811.8	0.22	72.0	137.3	0.91
S041	599.7	657.6	0.10	116.5	71.3	-0.39
S046	687.7	925.0	0.34	71.8	78.4	0.09
S048	353.9	551.9	0.56	25.4	24.2	-0.05
**B group average**	1054 ±644	1072 ±477	0.16	92 ±68	104 ±73	0.36

Discussion on microbiota and SCFA findings

The examination of individual bacterial ASVs identified 102 whose relative abundance changed over time and between treatment groups. The majority of these ASVs belonged to the family Lachnospiraceae, which, along with Ruminococcaceae and Bifidobacteriaceae, were identified as being part of bacterial co-abundance groups that are potentially beneficial in alleviating the social deficits of children with ASD [81]. The second and third clusters in the heatmap (Figure 3) display ASVs whose abundance appears to increase post-probiotic supplementation (treatment A) but not in the placebo group (treatment B), under the combined effect 'timepoint:treatment', five of which are Lachnospiraceae and one Ruminococcaceae. Another cluster of interest, at the bottom of cluster six, shows six ASVs which, again, increased post-probiotic supplementation and comprise members belonging to Lachnospiraceae, Lactobacillaceae, Streptococcaceae and Marinifilaceae (Figure 3). Both Lactobacillaceae and Streptococcaceae strains are present in Vivomixx. Marinifilaceae, specifically *Odoribacter* spp. (as assigned here), is a butyrate producing bacteria whose abundance in the gut microbiome has been linked to child metabolic health [82] and infant neurodevelopment [83], and has been reported to increase post-probiotic treatment in children with ASD and attention deficit hyperactivity disorder (ADHD) [57]. However, whether *Odoribacter* spp. plays a role in ASD is unclear.

Despite the abundance of three Lactobacillaceae ASVs increasing post-probiotic supplementation, the average relative abundance of the family did not show significant differences between treatment groups (Figure 4), and when examining

the relative abundance of this group in individual children (Table S3) no pattern was apparent compared to that observed for Bifidobacteriaceae. This may be because Lactobacillaceae strains are present in dairy (e.g., yoghurt) and other fermented foods, and as such can be acquired through diet outside of probiotic supplementation. Bifidobacteriaceae, on the other hand, showed a significant increase post-probiotic treatment (Figure 4). *Bifidobacterium* spp. is a beneficial symbiont of the human gastrointestinal tract and is associated with reducing the colonisation of pathogenic bacteria, imparting anti-carcinogenic, immunostimulatory and anti-diarrhoeal properties, alleviating symptoms of lactose intolerance, and lowering serum cholesterol levels [84]. Many of these benefits may be related to its ability to metabolise complex sugars, resulting in the production of SCFAs (e.g., lactic, acetic, propionic, and butyric acid) [85]. Children with ASD are reported to have a reduced faecal *Bifidobacterium* spp. load compared to neurotypical children, which is suggested to contribute to differences in neurotransmitter levels [49],[50]. Therefore, increasing faecal bifidobacteria could potentially improve behaviour symptoms in children with ASD [51]. In our study, of the six individual children with increased Bifidobacteriaceae abundance, four displayed a greater relative increase than the average of the placebo group (1.39), and two showed a substantial relative change increase of 68.71 and 1316.84 (Table S3). This result suggests that supplementation with Vivomixx can increase Bifidobacteriaceae load in children with ASD, though the degree of increase is individual-specific. While it is not possible to identify whether the increased bifidobacteria ASVs are those included in Vivomixx, future studies could include metagenomic sequencing to address this question. Furthermore, while samples were collected within the first week post-treatment, it could be valuable to include the number of post-treatment days prior to sample collection as the bifidobacteria administered in Vivomixx could be still in the stool samples within the first 24 hours but lost by day 7 post-treatment. There is no available evidence of the recovery time in stool samples post-cessation of treatment for this probiotic.

One of the secondary outcomes of this pilot RCT was to assess whether probiotic supplementation would alter faecal SCFA concentrations. In the human body, SCFAs play an essential role in the microbiome–gut–brain axis as signalling metabolites, reported to regulate catecholamine production and preserve neurotransmitter phenotypes after birth. [86]. Our results showed no difference in the concentration of faecal acetate, propionate, butyrate or valerate between probiotic and placebo treatment groups. There is conflicting evidence regarding the relationship between SCFAs and ASD, with studies both reporting elevated or reduced SCFA levels in children with ASD compared to neurotypical children [15, 87-88]). Probiotic supplementation is proposed to improve ASD symptoms by regulating the gut–brain axis through modulation of SCFA producing-bacteria in the gut microbiome [86]. A recent systematic review of RCTs involving probiotic supplementation (mostly *Bifidobacterium* and *Lactobacillus* strains) found that 53% of studies reported an increase of at least one SCFA, 17% reported a reduction, and 30% reported no difference following probiotic supplementation [89]. Specific to ASD, a study by Adams et al. [88] measured significantly lower levels of faecal acetate, propionate, butyrate and valerate in children with ASD using probiotic supplementation (i.e., any type of probiotic daily) compared to those without. Conversely, Wang et al. [90] demonstrated an increase in faecal acetate, butyric, and propionic acid following supplementation with combined probiotics (*Bifidobacterium* and *Lactobacillus*) and prebiotic fructo-oligosaccharides, which coincided with an improvement in the child autism treatment evaluation checklist (ATEC) scores in speech–language communication and sociability. These reported inconsistencies in changes to faecal SCFA levels are most probably related to differences in probiotic formulations and strains used, as well as the subject variation study protocols, host genetics and environmental factors including ethnicity.
